# Effects of obesity on lung volume and capacity in children and adolescents: a systematic review

**DOI:** 10.1016/j.rppede.2016.03.013

**Published:** 2016

**Authors:** Aline Dill Winck, João Paulo Heinzmann-Filho, Rafaela Borges Soares, Juliana Severo da Silva, Cristhiele Taís Woszezenki, Letiane Bueno Zanatta

**Affiliations:** a Universidade de Caxias do Sul (UCS), Caxias do Sul, RS, Brazil; b Pontifícia Universidade Católica do Rio Grande do Sul (PUC-RS), Porto Alegre, RS, Brazil; c Instituto Cenecista de Ensino Superior de Santo Ângelo (Iesa), Santo Ângelo, RS, Brazil

**Keywords:** Lung function tests, Lung volume measurements, Total plethysmography, Obesity, Pediatrics

## Abstract

**Objective:**

To assess the effects of obesity on lung volume and capacity in children and adolescents.

**Data source:**

This is a systematic review, carried out in Pubmed, Lilacs, Scielo and PEDro databases, using the following Keywords: Plethysmography; Whole Body OR Lung Volume Measurements OR Total Lung Capacity OR Functional Residual Capacity OR Residual Volume AND Obesity. Observational studies or clinical trials that assessed the effects of obesity on lung volume and capacity in children and adolescents (0-18 years) without any other associated disease; in English; Portuguese and Spanish languages were selected. Methodological quality was assessed by the Agency for Healthcare Research and Quality.

**Data synthesis:**

Of the 1,030 articles, only four were included in the review. The studies amounted to 548 participants, predominantly males, with sample size ranging from 45 to 327 individuals. 100% of the studies evaluated nutritional status through BMI (z-score) and 50.0% reported the data on abdominal circumference. All demonstrated that obesity causes negative effects on lung volume and capacity, causing a reduction mainly in functional residual capacity in 75.0% of the studies; in the expiratory reserve volume in 50.0% and in the residual volume in 25.0%. The methodological quality ranged from moderate to high, with 75.0% of the studies classified as having high methodological quality.

**Conclusions:**

Obesity causes deleterious effects on lung volume and capacity in children and adolescents, mainly by reducing functional residual capacity, expiratory reserve volume and residual volume.

## Resumo

**Objetivo:**

Avaliar os efeitos da obesidade sobre os volumes e as capacidades pulmonares em crianças e adolescentes.

**Fontes de dados:**

Trata-se de uma revisão sistemática, através das bases de dados Pubmed, Lilacs, SciELO e PEDro, por meio das seguintes palavras-chave: Plethysmography, Whole Body OR Lung Volume Measurements OR Total Lung Capacity OR Functional Residual Capacity OR Residual Volume AND Obesity. Foram selecionados estudos observacionais ou ensaios clínicos que avaliaram os efeitos da obesidade sobre os volumes e as capacidades pulmonares em crianças e adolescentes (0 a 18 anos), sem qualquer outra doença associada, nos idiomas inglês, português e espanhol. A qualidade metodológica foi avaliada através da Agency for Healthcare Research and Quality.

**Síntese dos dados:**

Dos 1.030 artigos, apenas quatro foram incluídos nesta revisão. Os estudos totalizaram 548 participantes, com predomínio do sexo masculino e tamanho amostral entre 45 e 327 indivíduos; 100% dos estudos avaliaram o estado nutricional através do IMC (escore-z) e 50% informaram os dados da circunferência abdominal. Todos demonstraram que a obesidade causa efeitos negativos sobre os volumes e as capacidades pulmonares, causa redução, principalmente, da capacidade residual funcional em 75% dos estudos, do volume de reserva expiratório em 50% e do volume residual em 25%. A qualidade metodológica variou entre moderada e alta, com 75% dos estudos classificados com alta qualidade metodológica.

**Conclusões:**

A obesidade causa efeitos deletérios sobre os volumes e as capacidades pulmonares em crianças e adolescentes, com redução principalmente da capacidade residual funcional, volume de reserva expiratório e volume residual.

## Introduction

Childhood obesity is currently a major public health problem and increases at an alarming rate in the world's population, including the Brazilian population.^1^ It is estimated that 150 million adults and 15 million children are obese.^2^ Recently, epidemiological data indicated that the prevalence of obesity in the United States is approximately 17% and affects about 12.7 million children and adolescents.^3^ In Brazil, some studies show that the prevalence of obesity ranges from 2.4 to 19.2%, affecting more the South and Southeast regions.^4^

According to the World Health Organization, obesity can be defined as an abnormal condition of body fat or excess fat tissue, which causes damage to the individual's health.^1^ Some situations or clinical conditions seem to be associated with its development, such as sedentary lifestyle, asthma, diabetes, hypertension, cardiovascular and respiratory diseases. Among these, the respiratory system deserves special attention, as excess weight brings direct changes to ventilatory mechanics.^1^^,^ 5 ^,^ 6

In recent decades, previous studies suggested that obesity causes a major change in the respiratory system, resulting in loss on thoracoabdominal synchronism.^7^ The increase in body weight causes limitation of diaphragmatic mobility and reduced back movement, with impaired pulmonary gas exchange and breathing pattern control.^7^^-^ 10 Moreover, excess adipose tissue is associated with increased inflammatory mediators and cytokines, which could alter the pulmonary airways of these subjects and contribute to the development of bronchial hyperreactivity.^11^

In addition to the abovementioned changes, the research on the subject points to the presence of important lung function alterations in children and adolescents with excess fat, including reduced forced expiratory volume in one second (FEV_1_), forced vital capacity (FVC) and forced expiratory flow between 25 and 75% of FVC (FEF_25-75%_).^12^^,^ 13

A systematic review published in 2012^14^ showed, through a critical analysis of five studies, that obesity leads to losses mainly in FEV_1_ and FVC. However, this study only evaluated the effects of body weight on spirometric variables.^14^ Taking into account that the spirometry test directly investigates obstructive pulmonary parameters and that the obesity factor seems to affect more the restrictive pattern,^1^^,^ 5 the importance of investigating, through plethysmography, the effects of body mass on lung volume and capacity in children is emphasized. Moreover, to date, the results are contradictory in terms of the impact of obesity on these pulmonary outcomes in samples of young individuals.^15^

Therefore, considering the increasing prevalence of obesity in the pediatric population, the effects of this chronic condition on ventilatory mechanics and the conflicting information on the impact of excess weight on lung volume, we feel the necessity to obtain more information on the topic. Thus, the aim of this review was to evaluate the effects of obesity on the lung volume and capacity in children and adolescents.

## Method

The study consists of a systematic review carried out through a search in the PubMed, Lilacs, SciELO and PEDro databases. Observational studies and clinical trials were selected in English, Portuguese and Spanish, without any filters for age and year of publication of the articles. The period of study selection was from September to October 2015.

The search used for the selection of articles was based on six key words associated with Boolean descriptors. The following strategy was used: plethysmography, whole body OR lung volume measurements OR total lung capacity OR functional residual capacity OR residual volume AND obesity. These descriptors should be included at least in the title, abstract or in the keywords.

Studies that evaluated lung function by plethysmography in obese children and adolescents (0-18 years) were used as inclusion criteria. Obesity was defined as body mass index (BMI) z-score values>+2 or ≥97th percentile. On the other hand, review studies, case reports, articles that did not assess lung volume and capacity through body plethysmography, studies that assessed respiratory function through other evaluation methods or did not normalize the results of pulmonary function (plethysmography) in z-scores, percentage of predicted or the ones that did not compare the results with those from a control group were excluded. Studies evaluating the adult age group or did not include only children and adolescents were also excluded. Moreover, studies with subjects with any other chronic diseases were excluded.

After identifying the descriptors in the title, abstract and/or keywords, the abstracts of selected articles were read to assess the adequacy according to the eligibility criteria. Studies that met the predetermined criteria had the full text read for detailed analysis and data extraction. Article search and analysis, according to the abovementioned strategy were carried out independently by two reviewers and disagreements were resolved with a third reviewer, by consensus.

The following characteristics of the studies were recorded: first author's name, year of publication of the study, country (source) of data collection, age range, sample size, body mass index (BMI), waist circumference, plethysmography equipment, methodology used, type of data presentation (z-score, percentage of predicted or liters), assessed plethysmographic variables [expiratory reserve volume (ERV), inspiratory reserve volume (IRV), residual volume (RV), functional residual capacity (FRC), vital capacity (VC), inspiratory capacity (IC) and total lung capacity (TLC)], extra pulmonary tests, main plethysmographic results and additional results.

Methodological quality was assessed by two reviewers and any divergences were resolved by consensus. An appropriate scale for observational studies by the Agency for Health Care Research and Quality (AHRQ) was used.^16^ This tool evaluates nine items related to the study subject, methodological aspects, consistency of results, discussion and sponsorship. The final sum of each evaluated item totals a score of 100, with the studies being classified as low (<50 points), moderate (50-66 points) and high (>66 points) methodological quality.

## Results

**Figure 1:**
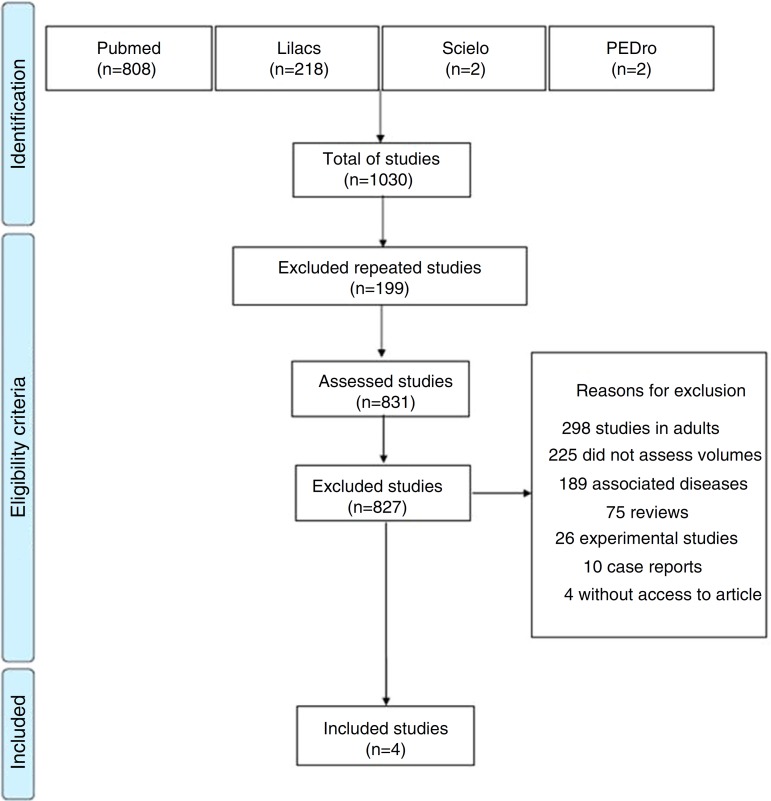
Flowchart of the total number of articles found in the databases.

A total of 1030 articles were identified, 808 in Pubmed, 218 in Lilacs, two in SciELO and two in PEDro. Of these, 199 were excluded for being repeated in the assessed databases and 827 because they did not meet the eligibility criteria of this review. Thus, only four studies were included, which evaluated the effects of obesity on lung volume and capacity in children and adolescents through body plethysmography. Fig. 1 shows a flowchart related to the total number of articles found in the assessed databases and reasons for study exclusion.

The selected studies totaled a sample of 548 participants, with a prevalence of 296 (54%) of male individuals. The sample size of the studies varied greatly, ranging from 45 to 327 subjects. The age of the selected participants ranged from 6 to 18.9 years. All studies evaluated BMI through z-score and two (50%) reported data from the waist circumference. Of the four studies, two were carried out in Asia, one in America and one in Europe (Table 1).

**Table 1:** Identification and characteristics of the studies included in this review.

First author	Year	Country	Age range (years)	Sample size	BMI (z-score)	Waist Circumference
Davidson et al.^17^	2014	Canada	6–17	327	2.18±0.41	NA
van de Griendt et al.^18^	2012	The Netherlands	8.5–18.9	112	3.38±0.40 (2.50–4.62)	122.2±15.7
Li et al.^19^	2003	China	7–18	64	2.42 (2.13–2.66)	NA
Kongkiattikul et al.^20^	2015	Thailand	8.6–17.3	45	3.2±0.5 (1.9–4.6)	99.1±10.4 (81–134)

BMI, body mass index; NA, information not available.

Regarding the characteristics of the equipment used to measure lung volume and capacity by plethysmography, three (75%) studies used the SensorMedics equipment and only one used a Jaeger device. Of the studies, three (75%) followed the recommendations of international guidelines, while one did not report the methodology used to obtain the pulmonary data. All studies included normalized the findings on lung volume and capacity through the percentage of predicted, although only one (25%) evaluated some plethysmographic variables in absolute values (liters). Additionally, all of them measured spirometric variables and 50% measured the carbon monoxide diffusion (DLCO).

The assessed plethysmographic data included VC, ERV, RV, FRC and TLC. All studies showed that obesity has a negative effect on lung volume and capacity, mainly reduced FRC in 75% of studies, of ERV in 50% and RV in 25%. Overall, the studies have also tested the associations of ventilatory variables and outcomes related to obesity, including BMI, waist circumference, fat mass index and waist-to-height ratio (Table 2).

**Table 2:** Characteristics and main results of the studies included in this review.

Type of equipment	Methodology used	Data presentation	Plethysmographic variables	Additional pulmonary tests	Main plethysmographic results	Additional results
*SensorMedics* 6200^17^	*American Thoracic Society* (ATS)	% of predicted and in liters*	TLC, VC*, FRC*, ERV*, RV*	Spirometry DLCO	Mean reduction of 0.44 of FRC, 0.22 of ERV and 0.20 of RV	Reduction of FEV_1_/FVC (*p*<0.001). Positive linear association between BMI and FVC (*p*<0.001) and DLCO (*p*=0.015) Negative linear association between BMI and FRC (*p*<0.001), ERV (*p*<0.001), RV (*p*<0,001) and FEV_1_/FVC (*p*<0.001).
*Jaeger Viasys* 18	NA	% of predicted	TLC, ERV, VC	Spirometry	Mean reduction of 14.3% in ERV	Increase of 2.91% (*p*=0.002) of FEV_1_, 3.08% (*p*=0.001) of FVC, 2.27% (*p*=0.001) of TLC, 14.8% (*p*<0.001) of ERV after reduction in body weight. Negative correlations of the increase in ERV with BMI (r=-0.27; *p*=0.01) and WC (r=-0.34; *p*=0.002).
*SensorMedics* 6200^19^	*British Thoracic Society*	% of predicted	TLC, RV, FRC	Spirometry DLCO	Mean reduction of 7% in FRC	Reduction of 23.5% in DLCO. Negative correlations with subtotal body and trunk fat, respectively, of: FRC (r=–0.367; *p*<0.01; r=-0.337; *p*<0.01), RV (r=-0.298; *p*<0.05; r=-0.290; *p*<0.05), TLC (r=-0.268; *p*<0.05; r=-0.264; *p*<0.05).
*SensorMedics* 6200^20^	*European Respiratory Society* (ERS)	% of predicted	TLC, FRC, RV	Spirometry	Mean reduction of 33.1% in FRC	Negative correlations between FRC with BMI (r=-0.32; *p*=0.03), waist-height ratio (r=-0.32; *p*=0.02), % body fat (r=-0.32; *p*=0.03),% fat in trunk (r=-0.32; *p*=0.04) and FMI (r=-0.36; *p*=0.02).

%, Percentage; VC, vital capacity; TLC, total lung capacity; FRC, functional residual capacity; RV, residual volume, ERV, expiratory reserve volume; FEV1, forced expiratory volume in one second; FVC, forced vital capacity; DLCO, carbon monoxide diffusion capacity; WC, waist circumference; FMI, fat mass index; NA, information not available.

Finally, regarding the methodological quality of the selected articles (Table 3), the average score was 71 points, ranging between 63 and 83. Of these, one (25%) study had a score compatible with moderate methodological quality and three (75%) with high quality, according to the AHRQ scale. Factors that lowered the quality score of the selected studies were associated to items related to the study population, comparison of subjects, outcome measurement, statistical analysis, discussion of data and information on support and/or funding of the study. It is noteworthy that of the four studies, only one had justification for the sample size, as well as reported information on blinding regarding when assessing the results. Furthermore, only two studies reported research support and funding, none of them calculated the power of analysis, as well as how potential confounders and result biases were assessed.

**Table 3:** Assessment of the methodological quality of the studies included in the systematic review.

Criteria assessed	Reference score	Davidson et al.^17^	van de Griendt et al.^18^	Li et al.^19^	Kongkiattikul et al.^20^
Question of the study	2	2	2	2	2
Study population	8	5	5	5	8
Comparability of individuals for the observational studies	22	16	14	11	11
Exposure or intervention	11	11	11	11	11
Outcome Measures	20	20	15	15	15
Statistical Analysis	19	12	9	7	7
Results	8	8	*8*	8	8
Discussion	5	4	3	4	4
Funding and sponsorship	5	5	0	0	5
**Total score**	100	83	67	63	71

## Discussion

In this review, four studies were identified, most of them with high methodological quality, which evaluated the effects of obesity on lung volume and capacity in children and adolescents.^17^^-^ 20 The findings showed a reduction mainly in FRC, ERV and RV, indicating negative effects of excess body weight on these pulmonary outcomes.

Studies carried out in obese individuals with no associated comorbidities indicate that the increase in adipose tissue in the chest and abdomen area causes an increase in the intra-abdominal pressure, with consequent reduction in lung compliance and chest wall mobility.^14^^,^ 15

These alterations result in thoracoabdominal asynchrony, lead to diaphragmatic contraction impairment due to increased load on the abdomen and also impair the increase in chest diameter through the movement of the ribs. Some findings suggest that the increased body mass is associated with higher tensile strength and decreased distensibility of extrapulmonary structures.^15^^,^ 21

Under normal physiological conditions, the FRC is the point of balance of elastic retractions between the lung and the chest wall.^22^ Thus, whereas FRC is the sum of RV and ERV, any change in these ventilatory variables implies in changes of this point of balance, as demonstrated by Davidson et al.^17^ Changes in pulmonary bellows cause reduced lung volumes, with subsequent establishment of a restrictive pattern.^23^^,^ 24 Kongkiattikul et al.^20^ also reported that the reduction of FRC leads to increased air resistance, peripheral vascular resistance and impairs alveolar ventilation. Furthermore, these data suggest that physiological changes increase the risk of atelectasis and hypoxia in obese individuals.^20^^,^ 25 A systematic review carried out in the obese adult population also showed reduction of the same plethysmographic variables, with the addition of TLC and VC.^15^

Half of the included studies was carried out in the Asian continent,^19^^,^ 20 followed by the American and European continents.^17^^,^ 18 To date, no study was found carried out in the Brazilian pediatric population that aimed to evaluate the influence of obesity on lung volume and capacity. Perhaps this lack of information at the national level is because there are few body plethysmographs in the country, as well as the high cost of the equipment and also the fact that it is harder to obtain a satisfactory test in a pediatric sample.^26^ Most studies used SensorMedics equipment^17^^,^ 19 ^,^ 20 and all of them normalized of lung volume results through the percentage of predicted,^17^^-^ 20 although a study did not report the equation used.^18^

Of the included studies, three followed international guidelines for body plethysmography, including the American Thoracic Society, European Respiratory Society and the British Thoracic Society.^17^^,^ 19 ^,^ 20 In general, the methods used seem to include the same ventilatory maneuvers to obtain lung volume and capacity, which contributes to greater external validity of the results.

Recently, a systematic review showed the negative effects of childhood obesity on spirometric variables, indicated a reduction in FEV_1_, FVC and FEV_1_/FVC ratio. However, this study did not investigate the effects of the obesity factor on lung volume and capacity.^14^ Taking into account that obesity may be considered a disease with a restrictive pattern,^1^^,^ 5 the data shown here add important information about the impact of the disease on children's respiratory system, compared to the study published in 2012.^14^ It is expected that future research, particularly at the national level, be designed to assess the effects of this chronic condition on the pulmonary system. However, we emphasize the importance that new studies stratify their age groups (children and adolescents), to investigate the possible influence of maturational state on these outcomes.

Moreover, it becomes essential to divide these subjects as to different degrees of obesity, as in the present review it was not possible to assess the influence of these factors. Although it is possible to evaluate the body mass of children and adolescents through several methods, BMI quantification stands out as a simple and low-cost tool.^27^ It is also known that normalization using percentiles or z-scores is essential for a better assessment of the nutritional status of the pediatric population.^27^^,^ 28 All studies included in this review evaluated BMI through z-scores, which is in accordance with pediatric recommendations.^27^^,^ 28 Of these, two studies^17^^,^ 20 showed the negative influence of BMI on FRC, ERV and RV through correlation testing and regression models. These results suggest that the higher the body composition, the lower the lung volume and capacity. Furthermore, previous studies have shown negative correlation between BMI and spirometric variables, including FEV_1_, FVC, FEF_25-75%_ and FEV_1_/FVC ratio.^10^^,^ 17 ^,^ 20 ^,^ 29 These findings support the hypothesis that increased body mass cause limitation in diaphragmatic mobility, reduction of costal movement, and impairs pulmonary physiology.^7^^,^ 10 Griendt et al., 18 demonstrated that body weight reduction is related to significant improvements in FEV_1_, FVC, TLC and ERV, indicating that the BMI reduction contributes to the increase in these outcomes. The authors explain these results due to the fact that, by reducing body weight, one increases the thoracic compartment space and improves lung compliance, thus facilitating ventilatory mechanics. The correlation between the increase in ERV and decreased waist circumference supports this rationale.^18^

Previous studies have observed a significant association between cardiovascular risk factors and waist circumference.^30^^,^ 31 During childhood and adolescence, excess body weight may be associated with relevant metabolic changes such as arterial hypertension, dyslipidemia and hyperinsulinemia, which results in metabolic syndrome.^32^^,^ 33 Previous data indicate that the association between obesity and metabolic syndrome is even stronger if adiposity is located in the abdominal region.^34^^,^ 35 In this review, two studies evaluated the waist circumference of its participants.^18^^,^ 20 Van Griendt et al.^18^ showed a negative correlation between the reduction in waist circumference and increased ERV. Kongkiattikul et al.,^20^ in addition to demonstrating a negative correlation between waist circumference and FRC, showed an inverse association between the waist-height ratio, body fat percentage and fat mass index with FRC. These results support the fact that the increase in adipose tissue in the abdominal area has a direct harmful effect to these individuals' pulmonary function.^20^ Other data also showed an inverse correlation of the waist-height ratio and anthropometric variables with pulmonary values.^36^ Of the two studies that evaluated the waist circumference, van Griendt et al.,^18^ showed higher values compared to the study of Kongkiattikul et al.,^20^ which can be attributed the fact that the first sample included older individuals (8.5 to 18.9 years) with severe obesity, weighing between 52.3 and 192.2kg.

Overall, the quality of the selected studies was high, ranging between moderate^19^ and high methodological quality.^17^^,^ 18 ^,^ 20 For this evaluation, we used a modified scale to quantify observational studies.^16^ This scale has shown to be adequate for this type of design^14^ and evaluates the research question, the study population, the comparison of participants, exposure measures, statistical analysis, results, discussion and funding.^16^ The studies included in this review seem to have some limitations, although it is believed that such restrictions did not influence the assessed research question, considering the good methodological quality that was attained.

Some factors lowered the quality score of the studies, such as the absence of sample calculation^17^^-^ 19 the power of analysis,^17^^-^ 19 the information regarding blinding in the quantification of results,^18^^-^ 20 the care regarding the possible confounding variables^17^^-^ 20 and the impact of biases on the results.^17^^-^ 20 As most of the studies included in the review had a cross-sectional design,^17^^,^ 19 ^,^ 20 none of them assessed subjects for a period of follow-up, except for the study of van de Griendt et al.,^18^ which analyzed the effect of a weight reduction program on pulmonary function parameters in obese children and adolescents. Additionally, none of the studies evaluated the effects of dose-response and few carried out multivariate models or analyses, multiple comparison tests^17^^,^ 18 ^,^ 20 and reported funding of the study.^17^^,^ 20 The abovementioned items directly collaborated to reduce the score in the assessed studies.

In summary, the findings of this review demonstrate the deleterious effects of obesity on the lung volume and capacity in children and adolescents, reducing mainly the functional residual capacity, expiratory reserve volume and residual volume. The results highlight the need to create strategic effective measures to fight childhood obesity through intervention programs to prevent or mitigate the negative impact of obesity on lung function in this population.
